# Spectral Imaging Experiments with Various Optical Schemes Based on the Same AOTF

**DOI:** 10.3390/ma14112984

**Published:** 2021-05-31

**Authors:** Vladislav Batshev, Alexander Machikhin, Alexey Gorevoy, Grigoriy Martynov, Demid Khokhlov, Sergey Boritko, Vitold Pozhar, Vladimir Lomonov

**Affiliations:** 1 Scientific and Technological Center of Unique Instrumentation Russian Academy of Sciences, Butlerova Str. 15, 117342 Moscow, Russia; batshev@bmstu.ru (V.B.); gorevoy.av@ntcup.ru (A.G.); martynov.gn@ntcup.ru (G.M.); khokhlov.dd@ntcup.ru (D.K.); boritko@ntcup.ru (S.B.); vitold@ntcup.ru (V.P.); 2 Laser and Optical-Electronic Systems Department, Bauman Moscow State Technical University (National Research University), 2-nd Baumanskaya Str. 5, 105005 Moscow, Russia; 3 Institute of Information Technologies and Computer Science, Moscow Power Engineering University (National Research University), Krasnokazarmennaya 14, 111250 Moscow, Russia; 4 Moscow Institute of Physics and Technology, National Research University, 9 Institutskiy per., Dolgoprudny, 141701 Moscow, Russia; 5 Federal Scientific Research Center “Crystallography and Photonics”, Russian Academy of Sciences, 119333 Moscow, Russia; yupisarev@yandex.ru

**Keywords:** spectral imaging, acousto-optic interaction, image quality, confocal scheme, collimating scheme

## Abstract

Spectral image filtration by means of acousto-optical tunable filters (AOTFs) has multiple applications. For its implementation, a few different optical schemes are in use. They differ in image quality, number of coupling components, dimensions and alignment complexity. To choose the optical system of AOTF-based spectral imager properly, many factors have to be considered. Though various schemes of acousto-optic (AO) filtration have been tested and discussed, their comparative analysis has not been reported up to now. In this study, we assembled the four most popular schemes (confocal, collimating, tandem and double-path) using the same AO cells and experimentally compared their main features. Depending on the application, each scheme may be the basis of compact cost-effective spectral imaging devices.

## 1. Introduction

AOTFs have become a popular tool for various hyperspectral imaging applications in biomedicine [1,2], agriculture [3,4], aerospace [5,6] and other fields. Based on anisotropic Bragg diffraction of wide-band light by ultrasound in crystalline media, these spectral elements provide a good combination of optical (high spectral and spatial resolution, wide tuning range, etc.) and technical (compactness, absence of moving parts, etc.) features [7].

The imaging capabilities of AOTF-based systems are defined by multiple factors. Most of them, e.g., geometry of AO interaction, shape of AO cell (AOC) and structure of ultrasound beam [8,9,10], have been examined and discussed in detail. Besides these AOTF characteristics, the optical scheme of AO spectral filtration has a strong influence on the features of AOTF-based images [11]. In practice, a few different schemes are used.

A typical AOTF configuration includes a single AOC located between two polarizers crossed with respect to each other [12]. More effective in terms of spectral resolution and residual image distortion are double AOTFs that may consist of tandem AOCs [13] or utilize a double light pass through a single AOC by means of optical feedback [14] and back reflection [15].

Moreover, there are three image formation methods common for all AOTFs: collimating, confocal and convergent [10]. Collimating (telescopic) scheme is the most common and the simplest one and provides high image quality and spectral resolution. However, the non-uniformity of the central wavelength of filtered light across the field of view results in a specific image spatio-spectral distortion [16]. Confocal telecentric scheme, which forms the image inside AOC, is free from this drawback but leads to visualization of the inhomogeneous ultrasound structure in the filtered image and to spectral resolution degradation [17]. Specific AO aberrations (distortion and chromatic image drift) are present in both schemes. Converging scheme is used quite rarely [18].

Tandem AOTF consisting of two identical AOCs provides higher spatial and spectral resolution, but at the same time, lower light transmission and larger dimensions. Normally, tandem AOTF utilizes a collimating scheme to avoid additional couplers necessary for the confocal one. To reduce the dimensions and price of a tandem AOTF, a single AOC operating in double-pass collimating scheme can be used [19].

All these optical schemes differ in image quality, number of coupling components, dimensions, alignment complexity and other features. For each specific task and for each AOC, it is necessary to optimize the optical system for AO image filtration. Though the schemes mentioned above have been already tested and discussed, their comparative analyses have not been reported up to now. We need to extract the characteristic features of each scheme by separating them from intrinsic features of AOC itself.

**Figure 1:**
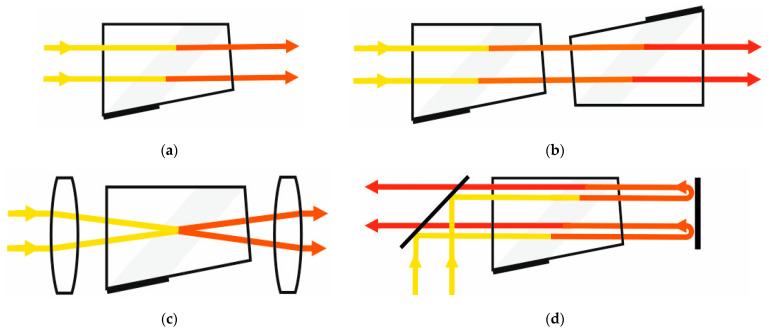
Typical schemes of AO filtration image: (**a**) single-crystal single-pass collimating scheme (S-coll); (**b**) double-crystal single-pass (tandem) collimating scheme (T-coll); (**c**) single-crystal single-pass confocal scheme (S-conf); (**d**) single-crystal double-pass collimating scheme (D-coll).

In this study, we assemble, test and analyze four various schemes (Figure 1). Two of them are commonly used schemes: collimating (coll) and confocal (conf) ones. Two others imply two-stage light diffraction: by two AOC in series (tandem configuration) and by the same AOC (double-pass configuration). All of them are described in Section 2. The comparison procedure and results are presented in Section 3, while the general conclusions are presented in the last section.

## 2. Materials and Methods

**Figure 2:**
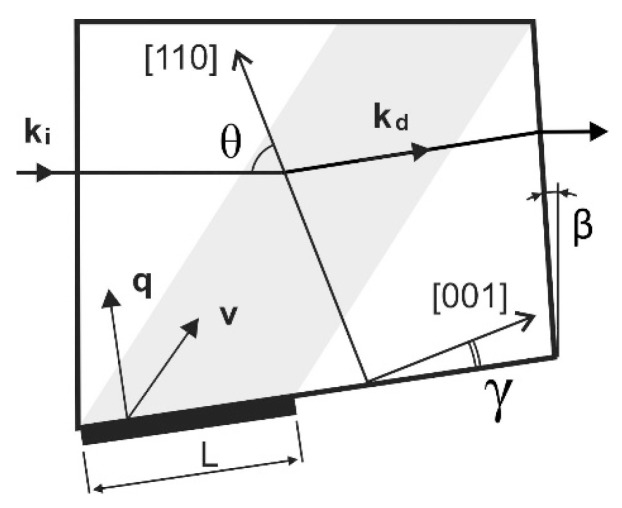
AO cell configuration. Group velocity **v** deviates from the ultrasound wave vector q due to walk-off effect; deflection of diffracted light wave vector k_d_ from incident k_i_ depends on θ and γ, while direction of output light beam is governed by back-facet inclination angle β. All the variants of experimental schemes have the same structure: object → input optics → AO spectral unit → output optics → photodetector.

In the experiments, we used a typical AOC made from paratellurite (α-TeO_2_), which is the most widely used AO uniaxial crystal. The geometry of this AOC is optimized for minimization of the chromatic image drift in the confocal optical scheme. It provides good spectral and spatial resolution and may be used in a wide spectral range [20]. Residual angular dispersion is 0.4°. The AOC has a cut angle γ = 7° (Figure 2). The wide-aperture diffraction geometry of e-polarized light [8,12] is realized for incident angle θ = 73.85°. Basic parameters: the piezotransducer length L = 12 mm, clear input aperture is 10 × 12 mm^2^, the angular aperture is 4° × 4°. An incident light beam must be directed normally to the front facet. The back-facet inclination angle is with respect to incident facet β = 2.3°. By varying the acoustic frequency within intervals of 60–110 MHz, one can tune the wavelength of filtered light in the range 450–850 nm. Driving acoustic power interval is 2.3–2.5 W. It is adjusted to equalize the AOTF’s light transmission in this range. In tandem AOTF two identical AOCs of this type was used.

**Figure 3:**
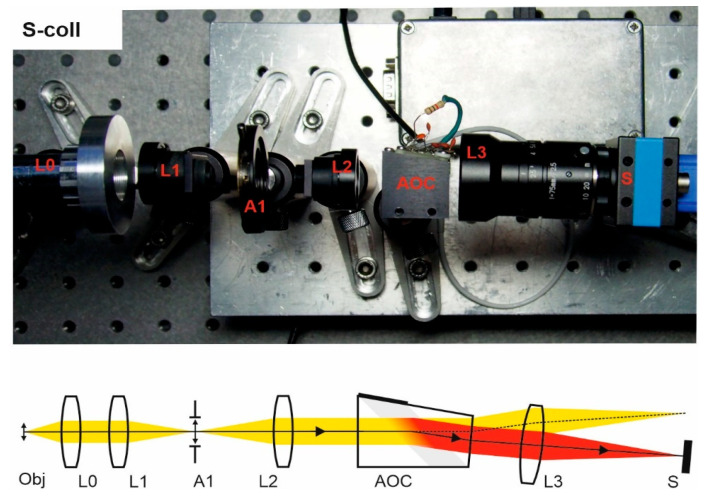
S-coll scheme. Breadboard model and optical beam-pass diagram.

**Figure 4:**
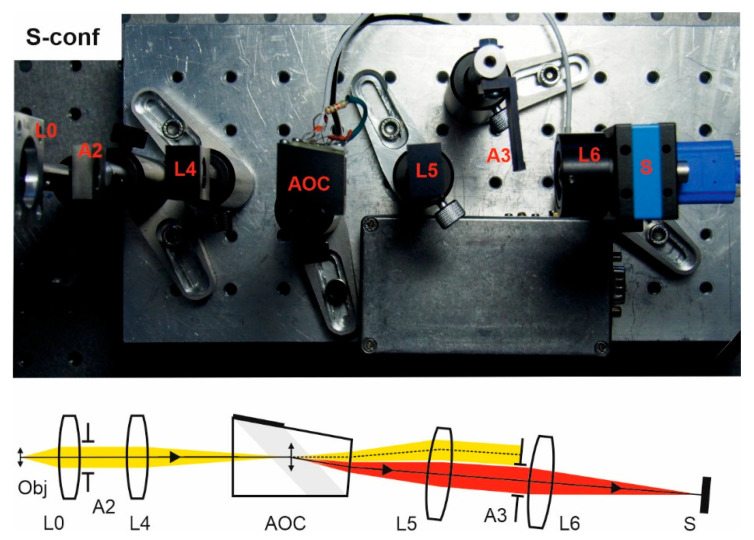
S-conf scheme. Breadboard model and optical beam-pass diagram.

**Figure 5:**
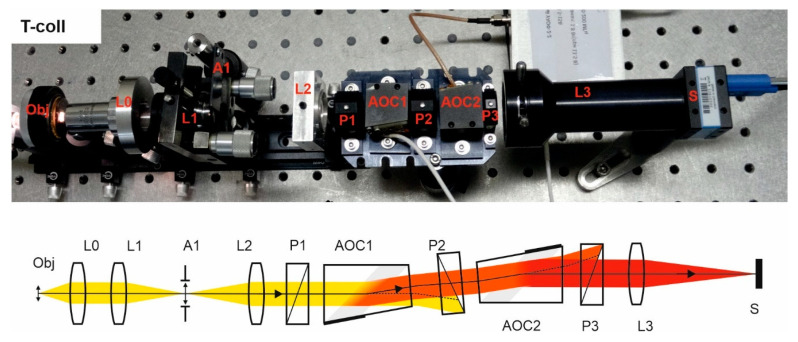
T-coll scheme. Breadboard model and optical beam-pass diagram.

**Figure 6:**
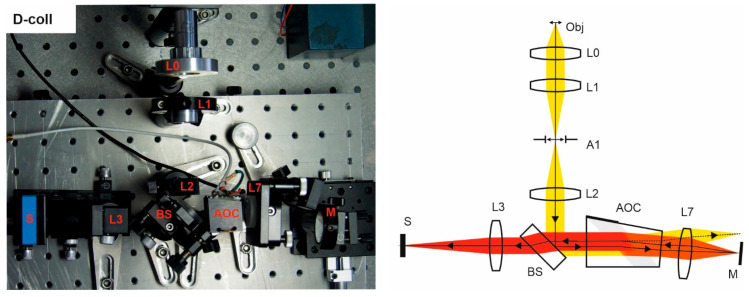
D-coll scheme. Breadboard model and optical beam-pass diagram.

In all the experiments presented below (Figure 3, Figure 4, Figure 5 and Figure 6), we used both standard and custom optical components. For image acquisition, the camera TheImagingSource DMK 37BUX178 with 1/1.8″ 6.3 MP CMOS sensor was installed. Depending on the inspected object, two different lenses L0 were utilized: an infinity-corrected microscope objective Carl Zeiss Planachromat 10/0.25 and photography lens Minolta MAXXUM 135/2.8. Afocal system L1, L2 consists of two identical standard lenses: 25 mm board lenses TheImagingSource TBL 25 or Thorlabs AC127-025-AB achromatic doublets. Focusing lens L3 is 75 mm Kowa LM75HC lens or Thorlabs AC127-075-AB achromatic doublet. Focusing lens in confocal scheme is 35 mm board lens TheImagingSource TBL 35. Coupling lenses L4 and L5 are 36 mm doublets specially designed and manufactured to compensate for the chromatic focal shift introduced by the AO cell in the confocal scheme. Lens L7 in the last setup is a 50 mm achromatic doublet.

A single-crystal single-pass collimating scheme (S-coll) is comprised of the following input optical part elements: input lens L0, field stop A1 inside the afocal system L1–L2, which define the collimated beam divergence preventing sensor irradiation by non-diffracted (unfiltered) light (Figure 3). An output lens L3 forms the spectral image on the sensor S.

Single-crystal single-pass confocal scheme (S-conf) comprises two similar optical systems in the input and output: a pair of lenses with intermediate diaphragm (Figure 4). The input system forms an intermediate image inside the AOC, while the output projects its filtered component onto the sensor S. The aperture stop A2 defines the convergence angle and output stop A3 blocks the non-diffracted (unfiltered) beam. Coupling lenses L4 and L5 are 36 mm custom-designed to compensate for the chromatic focal shift introduced by the AO cell. Other components are standard machine vision lenses.

Double-crystal single-pass (tandem) collimating scheme (T-coll) needs an afocal system L2–L3 in the input and output focusing lens L3 to transfer the object image into the sensor S (Figure 5). Tandem AOTF consists of two identical AO cells and three crossed Glan–Taylor calcite polarizers P1, P2, and P3. The second AO cell is rotated by 180° with respect to the first one. The polarizers are necessary to block the non-diffracted light beam leaving the filter at the same direction as the twice-diffracted one, whereas the lateral separation is insufficient due to the rather small deflecting angle. As the extinction ratio of polarizers is not always enough to eliminate non-diffracted light completely, we additionally utilized a field stop A1.

Since AO cells are identical and opposite turned, the same wide-aperture diffraction mode is realized in both AO cells, and, thus, image aberrations of distortion and chromatic drift are compensated. The drawback of the scheme is significantly increased energy losses. The advantages are higher spectral and spatial resolution.

Double-pass single-crystal collimating scheme (D-coll) contains beamsplitter BS, which divides it into three parts (Figure 6). The input optical system consists of a lens L0 and the afocal system L1-L2 with the field stop A1 inside. The spectral unit additionally comprises an AO cell, a retroreflector (lens L6 with the mirror M), which directs back the diffracted light emerging from AO cell, while the rest of the light does not return. The reflected spectrally selected light diffracts in the AO cell and is filtered one more time. In terms of ray tracing and spectral selection, the double-pass scheme is very similar to the tandem one with two identical AO cells.

In this scheme, we used a Thorlabs BSF10-A beamsplitter. A plate beamsplitter is preferable, as reflections from the edges of that usually degrade the image contrast. In general, the normal incidence on any flat surface located after BS should be avoided to protect the sensor from stray light that would reduce the image contrast. Even AR coating does not fully solve this problem, since the filtered light has a narrow spectrum and therefore its intensity is much less than the intensity of unfiltered light. To minimize stray light, the AOC is slightly tilted in the sagittal plane and its faces are coated with the antireflection coating. These measures, however, do not ensure solving the problem, because usually the tilt angle is small (otherwise it will cause optical aberrations, in particular astigmatism) and so reflections emerging in a wide spectral range make the selection of narrowband filtered light difficult. For this reason, polarizers are not suitable in this scheme. To solve the problem, we placed a field stop A1, like in the S-coll scheme (Figure 3), and thus have managed to adjust the scheme.

Generally, in this scheme, the transmission is significantly reduced by the beamsplitter, but there is an additional optical channel which can be used for white-light imaging or auxiliary purposes. Therefore, the double-pass scheme is promising for multimodal operation. One can also use an optional beamsplitter and other considered schemes to provide a similar multimodal operation. However, this will lead to additional losses in all other schemes.

## 3. Experimental Results

**Figure 7:**
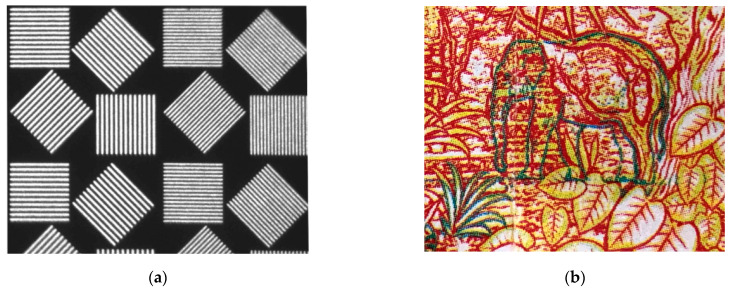
Spatial resolution test target (**a**) and color painting (**b**) used in the experiments.

Imaging capabilities of the described schemes were estimated with use of the test targets (Figure 7a) and specific color print (Figure 7b), for characterization of the spatial resolution, image distortion and spectral imaging contrast features.

For adequate comparison, one needs to equalize the image settings. To make the fields of view of all four systems roughly equal, we chose appropriate parameters of optical components L0–L6. The image longitudinal chromatic aberrations in the confocal scheme caused by the dispersion of the α-TeO_2_ crystal were compensated for by custom designed lenses L4 and L5. In collimating schemes, defocusing is negligible, and other optical elements were standard achromatic lenses.

**Figure 8:**
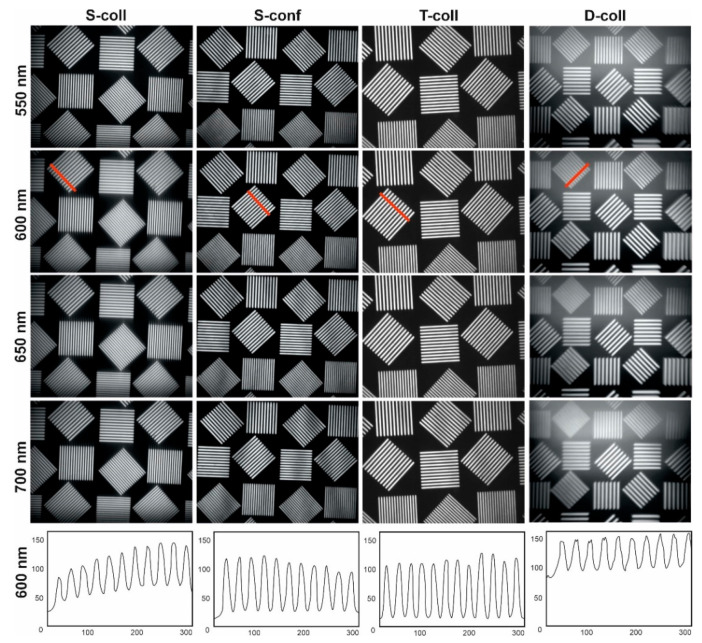
Spectral images of the test target and intensity cross sections along the red lines.

To equalize the image brightness, we optimized the intensity of the halogen light source as well as the exposure time and gain of the camera. For example, images at wavelength 600 nm (Figure 8) were recorded with the same lighting conditions, but different exposure times: 0.01 s (S-call and S-conf), 0.25 s (T-coll) and 0.5 s (D-coll).

The spectral resolution was measured in the central part of the field of view by the diffraction grating spectrometer Ocean Insight FLAME.

**Figure 9:**
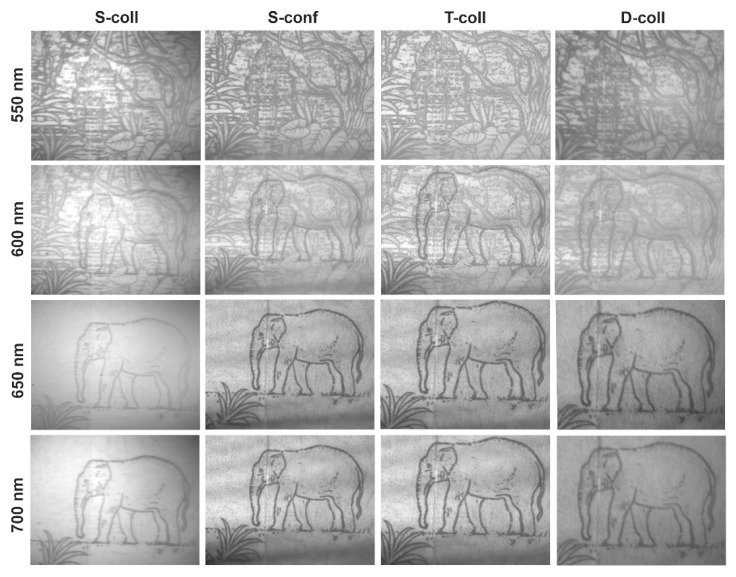
Spectral images of test color print recorded in four different schemes.

As can be seen from Figure 8 and Figure 9, classical single-crystal single-pass schemes (S-coll and S-conf) demonstrate rather similar features. The main differences are higher spatial resolution in the confocal scheme, with lower spectral resolution [17,18]. Double-stage schemes (T-coll and D-coll) exhibit significantly higher spectral and spatial resolution, in exchange for higher light losses [19]. The double-pass scheme (D-coll) combines the advantages of single-crystal and tandem AOTFs (high resolution at lower cost), but requires laborious adjustment. Generally, all the schemes demonstrate their usability so a given AO cell can be used in different schemes.

## 4. Discussion and Conclusions

In this experimental study, we assembled four popular optical schemes for AOTF-based spectral imaging (Figure 3, Figure 4, Figure 5 and Figure 6). The same typical AOCs operating in a conventional wide-aperture mode were exploited. In this way, we eliminated the influence of the AOC’s features and highlighted the differences caused by the optical scheme. Though all schemes are capable of providing acceptable image quality, there are considerable differences in spectral and spatial resolution and other imaging characteristics and, therefore, adequate choice of the optical scheme permits optimization of particular features important for solving the problem.

A detailed analysis of the obtained images (Figure 8 and Figure 9) is presented in Table 1. These results should be interpreted primarily as a comparative assessment of the studied schemes and not as a merit of the best performance achievable in each scheme.

The spectral resolution, as expected, is higher in schemes with double AO filtering (D-coll and T-coll). The best image contrast at the selected spatial frequency (~15 mm^−1^) is achieved in a scheme with two AOCs (T-coll) and in a confocal scheme. The worst contrast is demonstrated by the double-pass scheme (D-coll), in our opinion, for two reasons. First is stray light. Second, light energy losses make it necessary to work with long exposure times, and therefore, along with the signal, the background light is also exposed for longer.

In the experiments, we used AOCs with the geometry optimized for the confocal scheme. In particular, it compensates for the chromatic drift by the optimal output facet angle. Therefore, the chromatic drift is almost absent in S-conf, but it is noticeable in S-coll. In double-pass schemes, chromatic drift is compensated. Its residual non-zero values can be explained by inaccurate alignment. Exposure time in double-pass schemes is significantly higher due to double absorption in the AOC, a narrower spectral bandwidth, and the presence of a beamsplitter (in the D-coll scheme). In all schemes, the AOCs are the most expensive components, followed by polarizers. Thus, T-coll scheme seems to be the most cost ineffective.

This study paves the way to understanding the main issues related to the optical de-sign of AOTF-based spectral imagers. A good result is always a compromise between the AOTF configuration and the complexity of the optical coupling and adjustment. Depending on the application, each scheme may be effective. The study results are helpful for the development of new AOTF-based imagers. Each scheme is worth detailed studying and discussing in a separate article.

## Figures and Tables

**Table 1:** Experimental results.

Scheme	Spectral Resolution (at 600 nm), nm	Contrast at 15 mm^−1^	Chromatic (450–850 nm) Drift, %	Exposure Time (at 600 nm), s	Rel. Cost
S-coll	4.8	0.50	5%	0.01	1
S-conf	5.5	0.60	0.5%	0.01	1.1
T-coll	3	0.65	1%	0.25	2.5
D-coll	3.8	0.20	0.5%	0.5	1.5

## Data Availability

The data presented in this study are available on request from the corresponding author.
